# Effectiveness of a Wii balance board-based system (eBaViR) for balance rehabilitation: a pilot randomized clinical trial in patients with acquired brain injury

**DOI:** 10.1186/1743-0003-8-30

**Published:** 2011-05-23

**Authors:** José-Antonio Gil-Gómez, Roberto Lloréns, Mariano Alcañiz, Carolina Colomer

**Affiliations:** 1 Instituto Interuniversitario de Investigación en Bioingeniería y Tecnología Orientada al Ser Humano, Universitat Politècnica de València, C. Vera s/n, 46022 Valencia, Spain; 2 Servicio de Neurorrehabilitación de los Hospitales NISA Valencia al Mar y Sevilla Aljarafe, Fundación NISA, Valencia, Spain; 3 Ciber, Fisiopatología Obesidad y Nutrición, CB06/03 Instituto de Salud Carlos III, Av. Sos Baynat s/n, Univesity of Jaume I, 12071 Castellón, Spain

## Abstract

**Background:**

Acquired brain injury (ABI) is the main cause of death and disability among young adults. In most cases, survivors can experience balance instability, resulting in functional impairments that are associated with diminished health-related quality of life. Traditional rehabilitation therapy may be tedious. This can reduce motivation and adherence to the treatment and thus provide a limited benefit to patients with balance disorders. We present eBaViR (easy Balance Virtual Rehabilitation), a system based on the Nintendo^® ^Wii Balance Board^® ^(WBB), which has been designed by clinical therapists to improve standing balance in patients with ABI through motivational and adaptative exercises. We hypothesize that eBaViR, is feasible, safe and potentially effective in enhancing standing balance.

**Methods:**

In this contribution, we present a randomized and controlled single blinded study to assess the influence of a WBB-based virtual rehabilitation system on balance rehabilitation with ABI hemiparetic patients. This study describes the eBaViR system and evaluates its effectiveness considering 20 one-hour-sessions of virtual reality rehabilitation (n = 9) versus standard rehabilitation (n = 8). Effectiveness was evaluated by means of traditional static and dynamic balance scales.

**Results:**

The final sample consisted of 11 men and 6 women. Mean ± SD age was 47.3 ± 17.8 and mean ± SD chronicity was 570.9 ± 313.2 days. Patients using eBaViR had a significant improvement in static balance (p = 0.011 in Berg Balance Scale and p = 0.011 in Anterior Reaches Test) compared to patients who underwent traditional therapy. Regarding dynamic balance, the results showed significant improvement over time in all these measures, but no significant *group effect *or *group-by-time interaction *was detected for any of them, which suggests that both groups improved in the same way. There were no serious adverse events during treatment in either group.

**Conclusions:**

The results suggest that eBaViR represents a safe and effective alternative to traditional treatment to improve static balance in the ABI population. These results have encouraged us to reinforce the virtual treatment with new exercises, so an evolution of the system is currently being developed.

## Background

Acquired Brain injury (ABI) is the main cause of death and disability among young adults [1]. ABI can cause from mild to severe impairments in cognitive, motor or psychological functions leading to difficulties in familiar, vocational and social reintegration which diminishes health-related quality of life [2]. Among them, ABI can cause different levels of paralysis, such as hemiplegia or hemiparesis, which dramatically affect the balance control and, consequently, the performance of activities of daily living (ADL). Traditional balance training is based on the automatic repetition of specific movements. These methods can become repetitive and aimless, and thus reduce the motivation and adherence to treatment. Balance control, as the complex constellation of impairments following ABI, demands a multidisciplinary rehabilitation approach that, with the aid of new technologies, could maximize functional recovery.

In the last few years, there has been increasing research interest in the application of virtual reality (VR) technology to rehabilitation [3]. In contrast with traditional rehabilitation procedures, which may be tedious, resource-intensive and costly, VR provides patients with ABI opportunities to engage in meaningful, intensive, enjoyable and purposeful tasks related to real-life interests and ADL [4]. The published clinical results indicate that the recovery of motor function in ABI patients with motor difficulties appears to be enhanced by using VR technology [5-7]. Although most of these studies still consist of small experiments without randomized control trials [8], they demonstrate the feasibility of the application of VR technology in this clinical field.

In regard to standing balance, systems based on force platforms are particularly interesting, since they enable to estimate the weight distribution of the patients by means of pressure sensors [9,10]. These devices are expensive and require a dedicated area in the clinical facilities due to their size, weight and set-up. In this respect, computerized dynamic posturography can assess the ability of the automatic motor system to quickly recover from an unexpected external disturbance. Some of these systems even offer interactive and functional training exercises that fit the patients' conditions. In comparison with these platforms, the Nintendo^® ^WBB (a peripheral of the Nintendo^® ^Wii gaming system) is an inexpensive interface (less than $100USD) that has widespread availability. The WBB also has the advantage of being portable, easy and comfortable to handle thanks to its small size (0.511 m. wide by 0.316 m. long by 0.053 m. thick) and weight (3.5 kg. without batteries). Furthermore, it is a device with Bluetooth wireless connectivity that is battery operated. The WBB contains four force sensors (located in each corner) that are used to measure the user's center of balance and weight. Following the Nintendo^® ^gaming philosophy, users can interact naturally with the game (by means of weight transferences).

The number of studies that include Nintendo^® ^Wii or WBB in the rehabilitation process is increasing but still limited. Saposnik et al. evaluated the feasibility, safety and efficacy of VR rehabilitation using the Nintendo^® ^Wii gaming system with entertainment software to improve arm motor recovery in stroke patients [11]. Since the study is focused on improving arm recovery, the WBB was not used. Deutch et al. also use commercial software (Wii sports) to describe the feasibility and clinical efficacy of Nintendo^® ^Wii to augment the rehabilitation of an adolescent with cerebral palsy [12]. Loh et al. use this system as well and reported improvement in a group of patients with stroke in a non-controlled study [13]. Sugarman et al. report the feasibility and outcome of the WBB with a commercial program for balance training after stroke [14]. Although this software is not designed for balance recovery after stroke, they highlight its potential to be used in clinical settings in order to improve balance. In this sense, Clark et al. [15] demonstrated the convergent validity and the clinical utility of the WBB compared to a laboratory-grade force platform, which is considered the gold standard measure of balance. The results suggest that the WBB could be considered as a valid portable low-cost tool for assessing standing balance. However, the Nintendo^® ^Wii and WBB are entertainment systems oriented to healthy people that offer a gaming experience that differs from the therapy required by patients with ABI [16,17]. This fact has encouraged different authors to develop custom made applications oriented to diminished people using the WBB [18-22]. However, they are still very conceptual designs or lack more powerful studies to evaluate their efficacy.

Therefore, we designed eBaViR, a virtual rehabilitation system for balance recovery that provides motivational task oriented exercises specifically designed for ABI people by clinical therapists. The system can fit the patients' impairment to provide a particular training session, allowing the therapists to customize the duration and difficulty of exercises to the needs of the patients in each session.

The aim of this study is to evaluate the efficacy of the eBaViR system as a rehabilitation tool for balance recovery. In this contribution, we present a randomized and controlled single blinded trial to evaluate the influence of eBaViR on balance rehabilitation of ABI patients. We hypothesize that eBaViR is feasible, safe and potentially efficacious in enhancing standing balance.

## Methods

### Participants

Seventy-nine hemiparetic patients who had sustained an ABI and were attending a rehabilitation program were potential candidates for participation in this study. The inclusion criteria were: 1) age ≥16 years and <80 years; 2) chronicity > 6 months; 3) absence of cognitive impairment (Mini-Mental State Examination [23] cut-off >23); 4) able to follow instructions; 5) ability to walk 10 meters indoors with or without technical orthopaedic aids. The exclusion criteria were: 1) patients with severe dementia or aphasia; 2) patients whose visual or hearing impairment does not allow possibility of interaction with the system; 3) patients with hemispatial neglect; 4) patients with ataxia or any other cerebellar symptom.

After inclusion-exclusion criteria, a final consecutive sample of twenty patients remained from the total pool. This sample was divided into two groups according to the Berg Balance Scale score. Group A was made up of subjects with a high risk of falling, with Berg scores ranging from 30 to 45. Group B was made up of subjects with a low risk of falling, with a Berg score ≥46. All the subjects from both groups were randomly assigned to either a control group (traditional physiotherapy) or a trial group (eBaViR therapy). The randomization schedule was computer-generated using a basic random number generator. Two patients of the control group and one patient of the trial dropped out of the treatment due to causes unrelated to the study and system, and, consequently, their data are not included in the present contribution. The final sample consisted of 11 men and 6 women ranging from 16 to 76 years old (47.3 ± 17.8) and a mean chronicity of 570.9 ± 313.2 days. Etiology of acquired brain injury in this group of patients included severe traumatic brain injury (TBI) (n = 3), ischemic or hemorrhagic stroke (n = 11), and benign cerebral neoplasm (BCN) (n = 3). Table 1 shows a summary of the characteristics of the subjects.

**Table 1:** Characteristics of the participants

Issue	Control group	Trial Group	Significance
*Gender (n)*			NS (p = 0.858)
Male	5 (29.4%)	6(35.3%)	
Female	3(17.7%)	3(17.7%)	

*Age (years)*	49.13 ± 21.18	45.78 ± 15.38	NS (p = 0.704)

*Etiology (n)*			NS (p = 0.657)
Stroke	5 (29.4%)	6 (35.3%)	
TBI	1 (5.9%)	2 (11.8%)	
BCN	2 (11.8%)	1 (5.9%)	

*Time since injury (days)*	675.50 ± 283.11	478.00 ± 324.77	NS (p = 0.204)

The table shows the information of the patients. Age and time since injury are defined in terms of mean and standard deviation. Etiology is also expressed as a percentage of the total number of patients. NS: non-significant.

None of the participants had previous experience with virtual rehabilitation therapies. Written informed consent was obtained from patients for publication of this case report and accompanying images. A copy of the written consent is available for review by the Editor-in-Chief of this journal.

### Instrumentation

#### Hardware

The hardware components of the eBaViR system consist of a conventional PC, a 42" LCD screen and a WBB. The communication between this device and the computer is established via Bluetooth protocol. This way, the exercises run on a PC and the system uses the WBB as interface.

#### Software

As mentioned above, the eBaViR system does not use any commercial software. The exercises have been programmed using an authoring system for interactive 2D and 3D applications and designed with the help of clinical specialists in balance rehabilitation. The system has been developed with three main goals in mind: obtaining a valid and adaptive system for the balance rehabilitation of the patients, achieving a system that reinforces the motivation of the patients during the rehabilitative process and providing the therapists with objective data of the evolution of the patients.

The first goal is achieved thanks to the interface used in the system: the patient interacts with the games, specifically designed by specialists, through weight transferences in sitting and standing position, which is an essential process in standing balance rehabilitation [24,25]. The system calibrates the maximum excursion of the patients and adapts the range of motion of the exercises to fit their impairment. In addition, the therapist can configure the difficulty of the session with other parameters (number of items, speed, etc.). This way, the therapist can easily customize the training of each patient.

The second goal is achieved by designing the system with a playful scheme. For the patient, the system is basically a set of three games, which make the rehabilitative sessions more amenable. By means of its configuration, the system also tries to avoid frustrating gaming experiences in which the patients are not able to fulfill some tasks due to their motor or cognitive impairments.

The last goal is achieved registering the relevant outcomes of each exercise (scores, time, etc.) and the maximum excursion of the patients.

#### Use

In each session, the patient plays the three games of the system (*Simon*, *Balloon Breaker *and *Air Hockey*). A brief explanation of the purpose of each one of these games can be found in Table S1, Additional file 1. Although their visual aspect can be similar to commercial games, the games have been designed to optimize the visual and audio feedback and to simplify other stimuli to allow patients with cognitive impairments to follow the exercises and to focus on the motor task.

**Figure 1:**
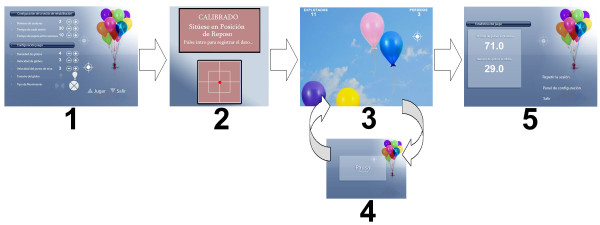
**Flow of the game**. The flow of the game can be divided into: 1) Setup; 2) Calibration; 3) Gameplay; 4) Break; 5) Scores.

The workflow is the same for the three games (see Figure 1).

First, each game begins with an initial setup screen (Figure 1, screen 1). This initial screen allows the therapist to parameterize the game to suit the needs of each patient. There are two sets of parameters: a set that configures the rehabilitation session (such as the duration of the session and the number of breaks per session), and a set that configures the game level (such as the size or the speed of the game elements).

ABI patients frequently have hemiparesis. One of the most characteristic consequences of incomplete recovery from hemiparesis is that there is a weight-bearing asymmetry in favour of the non paretic leg as well as increased spontaneous postural sway [26,27]. Because of this, the next step after configuration is to automatically adjust the sensitivity of the WBB to each patient's limitations (Figure 1, screen 2). The system is designed to record the centre of balance in the resting position of the patient and his/her range of motion in both the antero-posterior and medial-lateral planes. This provides the system with a very important advantage: it can adapt the games to the possibilities of each patient in each session.

**Figure 2:**
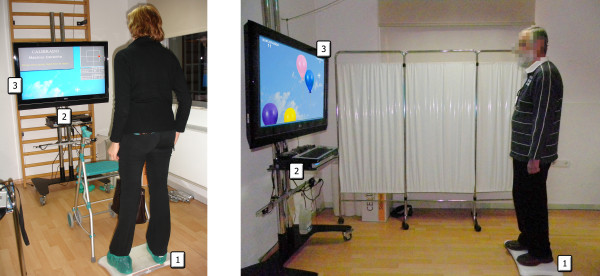
**Patients playing with the system**. The eBaViR system consists of: 1) a WBB; 2) a PC; 3) Video display. The eBaViR system is suitable for different levels of impairment and allows the treatment to be carried out in a secure way.

Once the rehabilitation session and the game have been configured and the virtual movements have been adjusted to the patient's excursion, the rehabilitation session can start. The eBaViR system allows the patient to play in standing or in sitting position to improve balance control in both conditions. In standing position the patients are required to maintain their soles on the WBB. In sitting position, the patients sit directly on the WBB. In the study presented in this paper, the patients play with the system in standing position (Figure 1, screen 3), with programmed pauses that are configured by the specialist in the setup screen (Figure 1, screen 4). During this session, the system gives the patient auditory feedback with a positive reinforcement when the patient accomplishes his/her goal throughout the sessions and a different reinforcement when the patient performs an incorrect action. The patient's score is continuously displayed and points are accumulated to calculate the final score. At the end of the rehabilitation session, the system shows the patient's percentage of hits and errors made during the game (Figure 1, screen 5). Game results and sounds serve as motivational elements. Figure 2 shows patients of the trial group in the course of a virtual rehabilitation session.

### Intervention

This clinical trial was carried out in a specialized neurorrehabilitation service of a large metropolitan hospital. Each patient participated in a total of 20 one-hour-sessions of rehabilitation and accomplished a minimum of 3 sessions and a maximum of 5 sessions per week. During control sessions, traditional rehabilitation exercises that focused on balance training were practiced either individually or in a group. The sessions of the trial group were programmed according to the protocol explained above.

All the patients were assessed before and after the rehabilitation program by a specialist who was blind to the patients' assignation. The clinical assessment considered clinical balance scales not only during static condition but also during dynamic tasks. On one hand, balance in static condition was assessed by the Berg Balance Scale (BBS) [28], the Brunel Balance Assessment (BBA) [29] and the Anterior Reach Test (ART) [30]. The ART measures the furthest distance that a person can reach forward while standing or sitting (without taking a step). On the other hand, balance in dynamic conditions was assessed by the Timed Stair Test (TST) [31], the Stepping Test (ST) [32], the 1-minute Walking Test (1MWT) [33], the 10-meter Walking Test (10MT) [34], the Time "Up and Go" Test (TUG) [35,36] and the 30-second Sit-to Stand Test (30SST) [37]. Table 2 describes these tests in more detail.

**Table 2:** Description of the balance scales and tests

Measure	Description
*Berg balance scale *(BBS)	A 14-item scale (measured from 0 to 4) designed to measure the balance of a person in a clinical setting. The scale ranges from 0 to 56.
*Brunel balance assessment *(BBA)	A functional balance measurement for people with a wide range of abilities; it has been specifically tested for post-stroke use in a clinical setting. The scale ranges from 0 to 14.
*Anterior reach test *(ART)	Maximum distance that the hand can be extended forward. The patient extends one arm to an angle of 90 degrees, while standing with legs about shoulder- width apart. The test can be run in sitting (ART-sit) or standing (ART-stand) position.
*Timed stair test *(TST)	Time required to climb and to descend five 8 cm high steps with handrails on both sides.
*Stepping test *(ST)	Times that the patient is able to place one foot onto a 7.5 cm high step and then back down to the floor within 15 seconds. The action is repeated as fast as possible during the test for the paretic and non-paretic leg. The step is placed 5 cm in front of the individual's feet.
*1-minute walking test *(1MWT)	Walking distance within a minute along an 8-shaped path that is 20 m long defined on a straight and nonslip surface.
*10-m walking test *(10MT)	Time to walk 10 m on a straight and nonslip surface.
*Time "up and go" test *(TUG)	Stop-watch measurement of the time taken for a return trip to a pole placed 5 m ahead, with the subject starting from a seated position in a chair.
*30-s sit-to-stand test *(30SST)	Repetitions of rising exercise from a sitting position on a bench.

A brief description of the scales and tests used in the study. The Berg and Brunel scales are widely used to assess the balance condition in a clinical setting. The tests are identified with the acronyms in brackets. The TST, 1MWT, 10MT, TUG, 30SST are commonly used in the literature. The ART and ST are defined above. The tests are identified with the acronyms in brackets.

In addition, a feedback questionnaire (SFQ) [38] was handed out to patients in order to obtain subjective information about the treatment. Therapists were also informally asked about the system.

### Data analysis

Demographical and clinical comparisons between groups were performed with independent sample t-tests and Chi-squared or Fisher exact tests, as appropriate. Repeated measures analyses of variance (ANOVAs) with time (before and after rehabilitation) as the within-subjects factor and treatment option (control versus trial) as the between-subjects factor were performed for each of the balance measures. The main effects of time, treatment option and the time-treatment option interaction effects were evaluated. Simple contrasts were conducted for each significant time main effect to determine the source of the significant difference. The α level was set at 0.05 for all analyses. All the analyses were computed with the Statview System for Macintosh, version 5.9 (SAS Institute Inc. 1992-98).

## Results

No significant differences in demographical (age and gender) or clinical (chronicity, etiology and laterality) variables at inclusion were detected between groups (Table 1).

A repeated measures ANOVA at the beginning and at the end of the clinical trial revealed a significant time effect for the BBS (p = 0.000), BBA (p = 0.048), standing ART (p = 0.005), ST-paretic (p = 0.021), ST-non paretic (p = 0.046), 1MWT (p = 0.007), TUG (p = 0.004) and 30SST (p = 0.003) (Table 3). No group effect was detected for any outcome, which confirms the comparability of both groups. Finally, significant group-by-time interaction was detected in the scores of the BBS (p = 0.011) and the ART in standing position (p = 0.011). With respect to these variables, post-hoc analysis showed better improvement in trial patients when compared to control subjects throughout the therapy.

**Table 3:** Clinical data

	**Before treatment **	**After treatment **	**Difference **	Significance
*BBS*				
Control	45.38 ± 7.35	46.88 ± 6.15	1.50 ± 1.31	T**(p = 0.000)
Trial	41.22 ± 10.57	45.44 ± 8.62	4.22 ± 2.33	GxT*(p = 0.011)
*BBA*				
Control	11.00 ± .1.31	11.13 ± 1.13	0.12 ± 0.35	T*(p = 0.048)
Trial	10.00 ± 2.00	10.33 ± 2.18	0.33 ± 0.50	
*ART standing (cm)*				
Control	25.44 ± 9.33	25.63 ± 9.74	0.19 ± 1.56	T**(p = 0.005)
Trial	24.13 ± 7.70	27.25 ± 10.38	3.12 ± 2.36	GxT*(p = 0.011)
*ART sitting (cm)*				
Control	40.06 ± 6.87	40.13 ± 7.66	0.06 ± 6.35	NS
Trial	34.83 ± 11.92	37.78 ± 12.34	2.94 ± 4.05	
*ST paretic (n)*				
Control	6.57 ± 2.30	7.57 ± 2.44	1.00 ± 1.29	T*(p = 0.021)
Trial	6.75+3.58	7.63 ± 4.00	0.87 ± 1.46	
*ST non-paretic (n)*				
Control	8.17 ± 1.72	9.50 ± 3.39	1.33 ± 1.97	T*(p = 0.046)
Trial	9.33 ± 2.81	10.50 ± 3.02	1.16 ± 1.83	
*TST (s)*				
Control	14.82 ± 9.42	12.13 ± 4.94	-2.69 ± 6.19	NS
Trial	15.38 ± 9.69	13.52 ± 9.60	-1.86 ± 4.67	
*1MWT (m)*				
Control	31.13 ± 13.59	36.38 ± 15.39	5.25 ± 3.99	T**(p = 0.007)
Trial	31.94 ± 12.47	42.69 ± 20.43	10.75 ± 13.78	
*10MT (s)*				
Control	14.57 ± 10.95	14.07 ± 9.02	-0.50 ± 2.16	NS
Trial	13.47 ± 8.29	13.47 ± 10.64	-0.00 ± 2.60	
*TUG (s)*				
Control	24.00 ± 14.87	19.52 ± 10.91	-4.48 ± 4.98	T**(p = 0.004)
Trial	20.99 ± 15.11	18.69 ± 13.43	-2.30 ± 2.33	
*30SST (n)*				
Control	6.88 ± 3.52	8.50 ± 3.12	1.62 ± 1.68	T**(p = 0.003)
Trial	7.56 ± 4.19	9.00 ± 4.74	1.44 ± 1.81	

Numerical data of the scores of scales and tests. The results are given in terms of mean () and standard deviation (σ). The subindexes 0 and 20 represent the assessments carried out before and after the treatment, respectively. G: group effect. T: time effect. GxT: group/time effect. * p < 0.05, ** p < 0.01. n represents the number of repetitions.

The mean ± SD SFQ score was 55.560 ± 5.940 over 65. In addition, all the patients remarked having had fun during the treatment. Only one case reported not being in control of the exercises. None of the patients suffered from spatial disorientation or cyber-sickness and no adverse symptoms were described by therapists.

## Discussion

Balance difficulties are amongst the most frequent motor disorders of ABI patients [39-41]. The recovery of this skill is an essential part of the rehabilitation process, since it is associated with a dramatic improvement in functional autonomy. The clinical study presented in this paper suggests that virtual rehabilitation provided significant improvement in static balance compared to traditional treatment. The homogeneity of the subject and their random assignment support the results.

According to BBS evolution, even though both groups show improvement over time, the results provide evidence of significant improvement in the trial group. As regards BBA evolution, the increase in BBA scores for both control and trial group is neglectable given de SD. Differences in the structure and content of the two measures may lead to this inconsistency; since the BBA score ranges from 0 to 14, this scale may not be as sensitive as the BBS to detect minor changes.

The results also showed a significant improvement of the sample in the ART over time with a better course of recovery for individuals in the trial group. According to the data, the trial group improved by 3 cm, which was considered a noticeable improvement. The functional reach test (FRT) has shown to improve over the course of rehabilitation, especially in the earlier stages of the recovery. Weiner et al. reported that the FRT is sensitive to changes in subacute patients undergoing traditional rehabilitation [42]. Brooks et al. described similar results in acute geriatric patients after a rehabilitation program [43]. Consequently, our results are promising considering the chronicity of our sample. In this sense, the FRT responsiveness for the experimental group was high (effect size: 1.3) compared to the control group (effect size: <0.1). Then, since this test is suggested to be a clinical measure of the stability and since it has correlation with balance abilities and risk of falls in the elderly [30,35], the clinical relevance of the improvement achieved during the present study should be considered.

Although the eBaViR system is designed to train static balance more specifically than dynamic balance, several outcome measures focused on balance abilities during gait and other complex motor tasks have also been considered. The results showed significant improvement over time in all these measures, but no significant *group effect *or *group-by-time interaction *was detected for any of them, which suggests that both groups improved in the same way. These results support the hypothesis that the system promotes the recovery of static balance, in which the system focuses on its exercises, while it has no significant effect in dynamic balance, since it is no specifically trained. Consequently, the therapy should be reinforced with dynamic exercises towards a comprehensive functional recovery, either through virtual or traditional therapy.

The significance of the *time effect *is especially outstanding due to the chronicity of the sample. The improvement in both groups is remarkable in spite of the fact that the chronicity (570.9 ± 313.2 days) is several times higher than a 6-month period, which is traditionally considered as the period with maximum recovery (where spontaneous recovery takes place) [44,45]. This fact makes the eBaViR achievements more relevant and suggests that forthcoming systems should have their basis more on the lost function and not so much on the chronicity.

With regard to feedback data, the SFQ score was high and corroborates the positive feedback of the patients who underwent the virtual treatment, and no cyber-sickness effect was detected. In addition, the therapists highlighted the ease and speed of use of the system.

In the last decade the emerging literature has demonstrated that training can lead to an enhancement of both the function and structure of the neural mechanisms. New rehabilitative strategies regarding motor learning and plasticity principles are focused on high-intensity, repetitive, and task-specific practice. According to these principles, virtual rehabilitation systems are excellent tools to enhance motor recovery since they allow repetitive intense training and on-screen observation, practice and representation of task-specific activities [46]. The eBaViR system has demonstrated to provide benefits to ABI patients affected by motor impairments and could be especially useful for balance rehabilitation under static conditions. In the field of skill learning and brain plasticity, the transfer of the skill acquired in the trained task to even other very similar tasks is generally the exception rather than the rule. This fact is well documented not only in the field of motor domain [47] but also in those processes involving perceptual learning [48] and cognitive recovery [49]. According to these principles, the greatest effects of training in our sample were observed in tasks that most closely mirror the trained task (static balance), with limited transfer of gains to other skills or to everyday competence (dynamic balance activities). Thus, these results encourage us to reinforce the virtual treatment with new exercises in order to promote improvement in the dynamic balance of patients. Future studies involving larger samples and including the aforementioned new exercises will be designed to evaluate the validity of these assumptions.

## Conclusions

eBaViR is a virtual rehabilitation system that uses the WBB with software that is specifically designed and developed in collaboration with clinical specialists for the rehabilitation of standing balance. This paper presents a single blinded study with two parallel groups. The study assessed the influence of a WBB-based virtual rehabilitation system (eBaViR) on standing balance rehabilitation with ABI patients and showed that virtual rehabilitation is capable of substantially improving the condition of the patients. However, interpretations of the results should be taken carefully considering the characteristics of our sample. The heterogeneous nature of acquired brain injury in our sample is also another limitation of the study.

The patients reported having had fun during the treatment without suffering from cyber side effects, which implies additional motivation and adhesion level to the treatment. Although no ergonomics test was considered, the specialists remarked on the ease and the speed of use. This makes it possible to spend most of the session on the treatment. As a token of their satisfaction, the therapists now continue using the eBaViR system on a daily basis for the standing balance rehabilitation of their patients and have encouraged us to add new exercises focusing on new issues. Currently, a second stage of eBaViR is being designed to incorporate all of their suggestions.

## Competing interests

The authors declare that they have no competing interests.

## Authors' contributions

JG, RL and MA contributed to the design of the study, the software development and the interpretation of the results. CC contributed to the design of the study, to the assessment of patients, to the acquisition of data and to its interpretation. All the authors have revised the manuscript and have given their final approval for publication.

## Supplementary Material

Additional file 1 **Table S1: Games developed for the eBaViR system**. Click here for file

## References

[B1] BC brain injury association http://www.bcbraininjuryassociation.com/acquired.php

[B2] Nichols-Larsen DS Clark PC Zeringue A Greenspan A Blanton S Factors influencing stroke survivors' quality of life during subacute recovery Stroke 2005 36 1480 1484 10.1161/01.STR.0000170706.13595.4f 15947263

[B3] Burdea GC Virtual rehabilitation-benefits and challenges Methods Inf Med 2003 42 5 519 523 14654886

[B4] Teasell R Meyer MJ McClure A Pan C Murie-Fernandez M Foley N Salter K Stroke rehabilitation: an international perspective Top Stroke Rehabil 2009 16 1 44 56 10.1310/tsr1601-44 19443347

[B5] Sveistrup H Motor rehabilitation using virtual reality J Neuroeng Rehabil 2004 1 10 10.1186/1743-0003-1-10 15679945 PMC546406

[B6] Holden M Virtual environments for motor rehabilitation: review Cyberpsychol Behav 2005 8 3 187 211 10.1089/cpb.2005.8.187 15971970

[B7] Cameirao MS Bermúdez S Verschure PFMJ Virtual reality based upper extremity rehabilitation following stroke: a review Journal of CyberTherapy & Rehabilitation 2008 1 1 63 7420662188

[B8] Crosbie JH Lennon S Basford JR McDonough SM Virtual reality in stroke rehabilitation: still more virtual than real Disabil Rehabil 2007 29 1139 1146 discussion 1147-1152 10.1080/09638280600960909 17613000

[B9] Haas BM Burden AM Validity of weight distribution and sway measurements of the Balance Performance Monitor Physiotherapy Research International 2000 5 1 19 32 10.1002/pri.181 10785908

[B10] Srivastavaa A Talyb AB Guptac A Kumarc S Murali T Post-stroke balance training: Role of force platform with visual feedback technique J Neurol Sci 2009 287 89 93 10.1016/j.jns.2009.08.051 19733860

[B11] Saposnik G Mamdani M Bayley M Thorpe KE Hall J Cohen LG Teasell R EVREST Steering Committee & EVREST Study Group for the Stroke Outcome Research Canada Working Group Effectiveness of Virtual Reality Exercises in STroke rehabilitation (EVREST): rationale, design, and protocol of a pilot randomized clinical trial assessing the Wii gaming system Int J Stroke 2010 5 47 51 20088994 10.1111/j.1747-4949.2009.00404.x PMC4880012

[B12] Deutsch JE Borbely M Filler J Huhn K Guarrera-Bowlby P Use of a low-cost, commercially available gaming console (Wii) for rehabilitation of an adolescent with cerebral palsy Phys Ther 2008 88 10 1196 1207 10.2522/ptj.20080062 18689607

[B13] Loh YJ Tjan SY Donald X Ernest T Chia PF Christopher KKW Kong KH A feasibility study using interactive commercial off-the-shelf computer gaming in upper limb rehabilitation in patients after stroke J Rehabil Med 2010 42 437 441 10.2340/16501977-0528 20544153

[B14] Sugarman H Weisel-Eichler A Burstin A Brown R Use of the Wii Fit system for the treatment of balance problems in the elderly: A feasibility study Virtual Rehabilitation International Conference 2009: 29 June-2 July, 2009; Israel: Haifa 2009

[B15] Clark RA Bryant AL Pua Y McCrory P Bennell K Hunt M Validity and reliability of the Nintendo Wii Balance Board for assessment of standing balance Gait Posture 2010 31 307 310 10.1016/j.gaitpost.2009.11.012 20005112

[B16] Lange BS Flynn SM Rizzo AA Initial usability assessment of off-the-shelf video game consoles for clinical game-based motor rehabilitation Phys Ther Rev 2009 14 5 355 363 10.1179/108331909X12488667117258

[B17] Deutsch JE Robbins D Morrison J Bowlby PG Wii-based compared to standard of care balance and mobility rehabilitation for two individuals post-stroke Virtual Rehabilitation International Conference 2009: 29 June-2 July, 2009; Israel: Haifa 2009

[B18] González-Fernández M Gil-Gómez JA Alcañiz M Noé E Colomer C eBaViR, Easy Balance Virtual Rehabilitation System: a Study with Patients Stud Health Tech Informat 2010 154 61 66 20543271

[B19] Young W Ferguson S Brault S Craig C Assessing and training standing balance in older aldults: A novel approach using the 'Nintendo Wii' Balance Board Gait Posture 2011 33 303 305 10.1016/j.gaitpost.2010.10.089 21087865

[B20] Lange B Flynn S Proffitt R Chang CY Rizzo A Development of an interactive Game-based rehabilitation tool for dynamic balance training Top Stroke Rehabil 17 5 345 352 10.1310/tsr1705-345 21131259

[B21] Shih CH Shih CT Chiang MS A new standing posture detector to enable people with multiple disabilities to control environmental stimulation by changing their standing posture through a commercial Wii Balance Board Res Dev Disabil 2010 31 1 281 286 10.1016/j.ridd.2009.09.013 19850444

[B22] Shih CH Shih CT Chu CL Assisting people with multiple disabilities actively correct abnormal standing posture with a Nintendo Wii balance board through controlling environmental stimulation Res Dev Disabil 2010 31 4 936 942 10.1016/j.ridd.2010.03.004 20381997

[B23] Folstein MF Folstein S Mchugh PR Mini-Mental State: a practical method for grading the cognitive state of patients for the clinicians J Psychiatr Res 1975 12 3 189 198 10.1016/0022-3956(75)90026-6 1202204

[B24] Cook A Anson D Haller S Postural sway biofeedback: its effect on reestablishing stance stability in hemiplegic patients Arch Phys Med Rehabil 1980 69 6 395 400 3377664

[B25] Lee WA Deming L Sahgal V Quantitative and clinical measures of static standing balance in hemiparetic and normal subjects Phys Ther 1988 68 970 976 3375321 10.1093/ptj/68.6.970

[B26] Geurts ACH de Haart M van Nes IJ Duysen J A review of standing balance recovery from stroke Gait Posture 2005 22 267 281 10.1016/j.gaitpost.2004.10.002 16214666

[B27] Pérennou D Weight bearing asymmetry in standing hemiparetic patients J Neurol Neurosurg Psychiatry 2005 76 5 670 678 10.1136/jnnp.2004.046565 15834015 PMC1739628

[B28] Berg K Wood-Dauphinee S Williams JI Maki B Measuring balance in the elderly: validation of an instrument Canadian Journal of Public Health 1992 83 2 7 11 1468055

[B29] Tyson SF DeSouza LH The Brunel balance assessment: a new measure of balance disability post-stroke Physiotherapy 2002 88 11 700 10.1191/0269215504cr744oa 15573837

[B30] Duncan PW Weiner DK Chandler J Studenski S Functional reach: a new clinical measure of balance J Gerontol 1990 45 6 192 197 10.1093/geronj/45.6.m192 2229941

[B31] Perron M Malouin F Moffet H Assessing advanced locomotor recovery after total hip arthroplasty with the timed stair test Clin Rehabil 2003 17 7 780 786 10.1191/0269215503cr696oa 14606746

[B32] Hill KD Bernhardt J McGann AM Maltese D Berkovits D A new test of dynamic standing balance for stroke patients: reliability, validity and comparison with healthy elderly Physiother Can 1996 48 257 262 10.3138/ptc.48.4.257

[B33] McDowell BC Kerr C Parkes J Cosgrove A Validity of a 1 minute walk test for children with cerebral palsy Dev Med Child Neurol 2005 47 11 744 748 10.1017/S0012162205001568 16225737

[B34] van Hedel HJ Wirz M Dietz V Assessing walking ability in subjects with spinal cord injury: validity and reliability of 3 walking tests Arch Phys Med Rehab 2005 86 2 190 196 10.1016/j.apmr.2004.02.010 15706542

[B35] Podsiadlo D Richardson S The time "up & go": a test of basic functional mobility for frail elderly persons J Am Geriatr Soc 1991 39 2 142 148 1991946 10.1111/j.1532-5415.1991.tb01616.x

[B36] Steffen TM Hacker TA Mollinger L Age- and gender-related test performance in community-dwelling elderly people: six-minute walk test, Berg balance scale, timed up & go test, and gait speeds Phys Ther 2002 82 2 128 137 11856064 10.1093/ptj/82.2.128

[B37] O'Shea S Taylor N Paratz J Reliability of hand-held dynamometry and functional strength tests for the lower extremity in children with cerebral palsy Arch Phys Med Rehabil 2007 88 1 32 36 10.1016/j.apmr.2006.10.002 17207672

[B38] Kizony R Katz N Rand D Weiss PL A Short Feedback Questionnaire (SFQ) to enhance client-centered participation in virtual environments Proceedings of 11th Annual CyberTherapy 2006 Conference: Virtual Healing: Designing Reality: 12-15 June, 2006; Canada: Gatineau 2006

[B39] Tyson SF Hanley M Chillala J Selley AB Tallis RC The relationship between balance, disability, and recovery after stroke: predictive validity of the Brunel balance assessment Neurorehabil Neural Repair 2007 21 341 346 10.1177/1545968306296966 17353462

[B40] Hammer A Nilsagård Y Wallquis M Balance training in stroke patients - a systematic review of randomized, controlled trials Adv Physiother 2008 10 163 172 10.1080/14038190701757656

[B41] Lubetzky-Vilnai A Kartin D The Effect of Balance Training on Balance Performance in Individuals Poststroke: A Systematic Review Journal of Neurologic Physical Therapy 2010 34 3 127 137 20716987 10.1097/NPT.0b013e3181ef764d

[B42] Weiner DK Bongiomi DR Studenski SA Duncan PW Kochersberger GG Does functional reach improve with rehabilitation? Arch Phys Med Rehab 1993 74 8 796 799 10.1016/0003-9993(93)90003-S 8347063

[B43] Brooks D Davis AM Naglie G Validity of 3 Physical Performance Measures in Inpatient Geriatric Rehabilitation Arch Phys Med Rehabil 2006 87 1 105 110 10.1016/j.apmr.2005.08.109 16401447

[B44] Jørgensen HS Nakayama H Raaschou HO Vive-Larsen J Støier M Olsen TS Outcome and time course of recovery in stroke. Part II: Time course of recovery. The Copenhagen Stroke Study Arch Phys Med Rehabil 1995 76 5 406 412 10.1016/S0003-9993(95)80568-0 7741609

[B45] Ferrarello F Baccini M Rinaldi LA Cavallini MC Mossello E Masotti G Marchionni N Di Bari M Efficacy of physiotherapy interventions late after stroke: a meta-analysis J Neurol Neurosurg Psychiatry 2011 82 136 143 10.1136/jnnp.2009.196428 20826872

[B46] Dobkin BH The clinical science of neurological rehabilitation Oxford University Press 2003 Chapter 3 2

[B47] Rieser JJ Pick HL Jr Ashmead DH Garing AE Calibration of human locomotion and models of perceptual-motor organization J Exp Psychol Human 1995 21 480 497 10.1037//0096-1523.21.3.480 7790829

[B48] Karni A Sagi D Where practice makes perfect in texture discrimination: Evidence for primary visual cortex plasticity P Natl Acad Sci USA 1991 88 4966 4970 10.1073/pnas.88.11.4966 PMC51788 2052578

[B49] Ball K Berch DB Helmers KF Jobe JB Leveck MD Marsiske M Effects of cognitive training interventions with older adults: A randomized controlled trial JAMA-J Am Med Assoc 2002 288 18 2271 2281 10.1001/jama.288.18.2271 PMC2916176 12425704

