# Supplementary material for: Effectiveness of a Wii balance board-based system (eBaViR) for balance rehabilitation: a pilot randomized clinical trial in patients with acquired brain injury
Source: J Neuroeng Rehabil. 2011 May 23;8:30. doi: 10.1186/1743-0003-8-30 (PMC3120756; doi:10.1186/1743-0003-8-30)
Supplement: Additional file 1 — Table S1: Games developed for the eBaViR system. [file 1743-0003-8-30-S1.DOC]

## Additional file 1.

*Table S1*. Games developed for the eBaViR system

| **Game** | **Snapshot** | **Target** | **Interface** | **Movement** |
| --- | --- | --- | --- | --- |
| *Simon* | 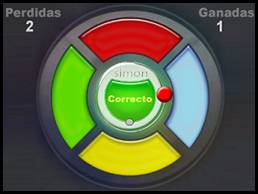 | To repeat the color sequence indicated by the system. | The patient moves a red circle to the next color of the sequence. | Medial-lateral and antero-posterior |
| *Balloon Breaker* | 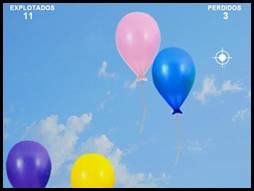 | To break the maximum number of balloons | The patient moves a crosshair towards the balloons; when it makes contact with a balloon, the balloon breaks. | Free |
| *Air Hockey* | 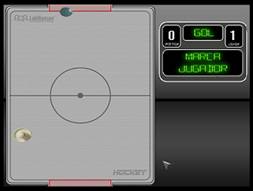 | To score goals in the opponent’s goalpost while defending one’s own goalpoast. | The patient moves the goalie; when it makes contact with the puck, the puck changes its trajectory. | Medial-lateral |
